# Reverse shoulder arthroplasty for two-parts proximal humerus fractures with “shish-kebab” technique

**DOI:** 10.1016/j.xrrt.2024.05.005

**Published:** 2024-05-25

**Authors:** Paolofrancesco Malfi, Roberto de Giovanni, Alessio Bernasconi, Valentina Rossi, Riccardo Grasso, Andrea Cozzolino

**Affiliations:** Department of Public Health, Orthopedic Unit, "Federico II" University, Naples, Italy

**Keywords:** Proximal humerus fracture, Two parts fracture, Surgical neck, Reverse shoulder arthroplasty, Tuberosity osteotomy, Shishkebab

Two-parts proximal humerus fractures (PHFs) can be classified as nondisplaced or with minimal displacement, which typically receive conservative treatment, and displaced (>1 cm, >45° according to Neer criteria), for which there is still no consensus regarding optimal treatment in the literature. Displaced two-parts PHFs account for 12.7% of all fractures of the proximal humerus^3^ and are associated with osteoporosis and poor bone quality.^27^

Historically, displaced two-parts fractures have been surgically treated with osteosynthesis either by open reduction and internal fixation (ORIF) or endomedullary nailing.^17^ Since the fracture line does not affect the tuberosities or the joint surface, fracture healing could be theoretically obtained without any joint disfunction.

The use of reverse shoulder arthroplasty (RSA) for the treatment of PHFs associated with poor bone quality has been introduced in 2007 by Walch et al.^30^ The most common indications for RSA are displaced three and four-parts fractures in elderly patients, associated glenohumeral joint dislocation and previous rotator cuff tear (RCT).^24^ A metanalysis conducted by Suroto et al,^29^ comparing RSA and ORIF for the treatment of three and four parts fractures, showed better clinical outcomes and less revision surgery in case of RSA.

On the contrary, there are a few studies looking at the use of RSA in case of surgical neck (SN) fracture. Reasons for the limited use of RSA in two parts PHFs are: the frequent need for a tuberosity osteotomy, a more challenging evaluation of the stem’s height and the use of longer cemented stems.

The aim of this study is to present a surgical technique to improve the implant of an RSA in two parts PHFs, avoiding tuberosity osteotomy and preserving the metaphyseal bone stock.

## Anatomy

The proximal humerus has been divided by Codman in four parts: humeral head (HH), lesser tuberosity (LT), greater tuberosity (GT), and SN. The anatomical neck separates the HH from the tuberosities, while the SN distinguishes the tuberosities from the diaphysis. The SN fractures are the most common type of two-parts fractures, while the anatomical neck ones are quite rare. The crucial anastomosis between the anterior and posterior circumflex humeral arteries (branches come from the axillary artery), which is formed around the SN of the humerus, provides vascularization to the proximal humerus.^20^ While it was initially thought that the anterior circumflex humeral artery was the primary source of blood supply to the HH, recent studies showed that this role is carried out by the posterior humeral circumflex artery.^12^

Since SN is in strict contact with the anterior and posterior circumflex arteries and the axillary nerve, neurovascular dysfunctions are not rare complications of two-parts fractures, especially in case of fragment displacement.

**Figure 1:**
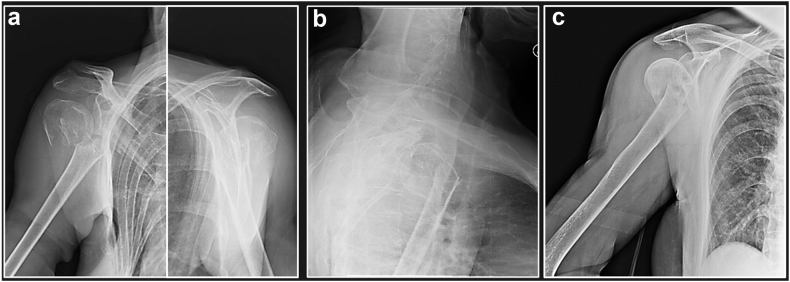
Different patterns of displacement of two-parts proximal humerus fractures: medial (**a**) and anterior (**b**) from Pec. Major traction; lateral (**c**) due to deltoid traction.

There are four main displacing forces relevant to two parts PHFs: 1. Pectoralis major (displaces humeral shaft antero-medially), 2. Deltoid (displaces humeral shaft posterolaterally), 3. Supraspinatus, Infraspinatus, Teres minor (displace and lateral/externally rotate the head), 4. Subscapularis (internally rotates the articular segment and/or LT). Several pattern of displacement can be observed according to the level of the fracture line, the integrity of musculotendinous units, and the trauma direction (Fig. 1).

## Indications and contraindications

Two parts fractures are indication for surgery according to: the severity of displacement, the risk of avascular necrosis, the bone quality, the presence of a fracture dislocation and/or a RCT and/or a symptomatic glenohumeral osteoarthritis. The specific amount of fragment displacement suitable for surgical treatment has been discussed in several papers. Neer defined 45° of fragments angulation and 1 cm of separation as the threshold for displacement.^21^ However even the same author recognized these parameters as arbitrary.^22^ Foruria AM et al recognized that the displacement of two part SN fractures usually consists in an anteromedial dislocation of the shaft, which could create mechanical impingement against the scapula or loose of bone contact with the HH.^5^ We believe that these two aspects could me more appropriate to indicate a surgical treatment rather than an arbitrary measure of displacement. While displacement is the key factor for surgical indication, the choice between osteosynthesis and replacement is linked to the risk of avascular necrosis, bone quality and the likelihood of complication associated with an ORIF through plating or nailing. Calcar length and comminution, medial hinge integrity, height of the HH fragment, absence of active back-bleeding from the HH and cortical index have all been proposed as prognostic factors to predict outcome after proximal humeral ORIF.^9^^,^^7^^,^^28^ However, there is no evidence that a single specific factor could predict a synthesis’ failure. On the contrary, the presence of massive RCTs or of osteoarthritis represents a good indication for RSA also in case of two part fractures even in case of relative young active patients. Preoperative imaging with plain radiographs and computed tomography should be performed to evaluate all these factors before surgery.

Beside fracture pattern, patients’ expectation, comorbidities and preinjury level of daily life activities should be considered. In case of low-demand patients, low compliance with rehabilitation protocol, severe comorbidities, conservative treatment should be preferred. The proposed surgical technique can be used only in cases of SN fracture. The proximal humeral metaphysis must be intact.

All general and local complications should be discussed with the patient, and the risk-to-benefit ratio should be carefully evaluated before surgery.

Clinical examination should rule out axillary nerve palsy, deltoid dysfunction, and evaluate integrity of the skin. Conservative treatment plays a role in cases where surgery is contraindicated, informing the patient of the possible functional limitations secondary to malunion or nonunion.

## Technique

**Figure 2:**
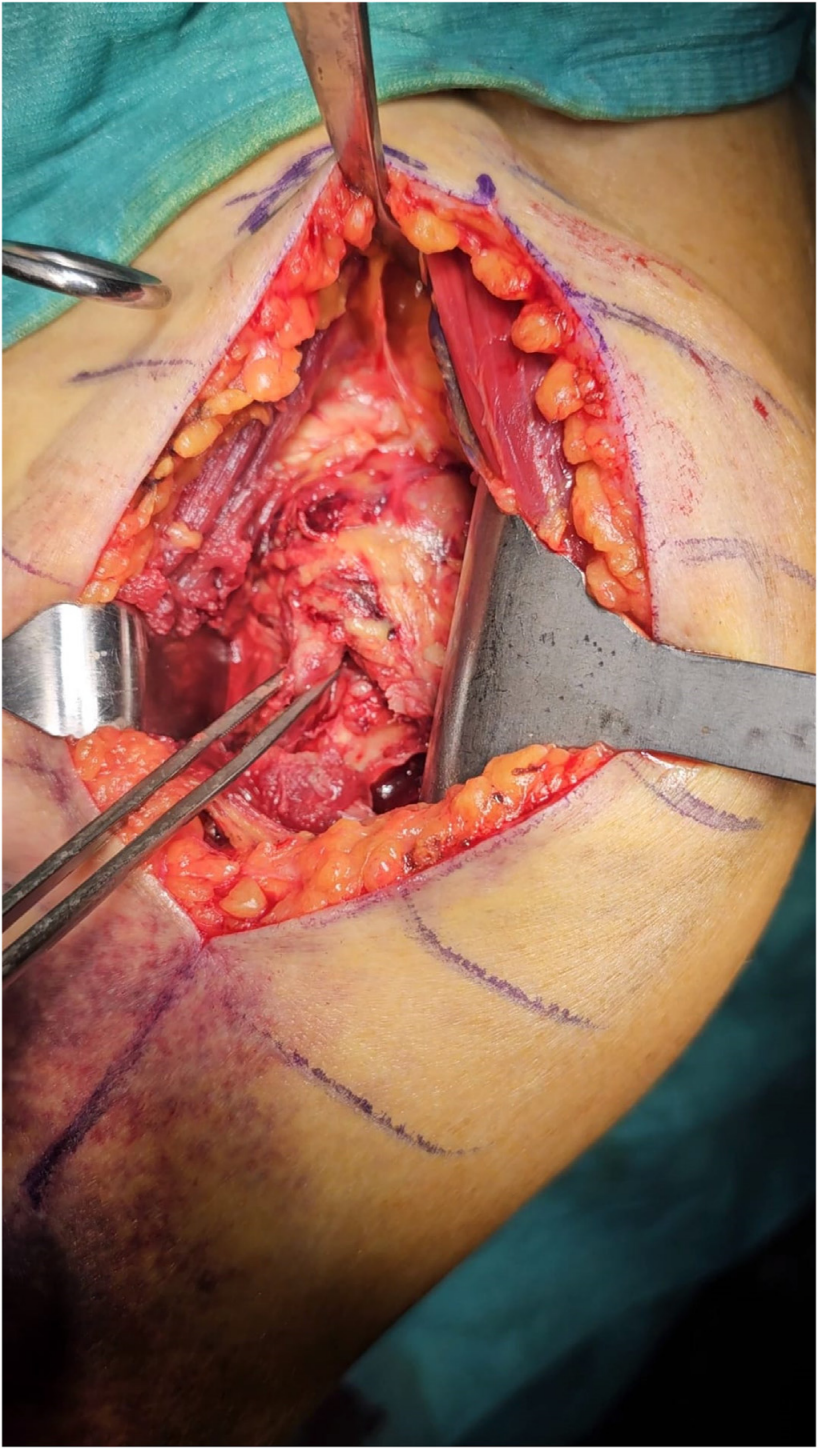
The LHB (caught in clamp) is identified and represents the main landmark for rotation of proximal fragments. *LHB*, long head of the bicep.

The patient is positioned in beach chair with the arm placed off to the side of the bed to facilitate humeral extension and adduction. After a standard deltopectoral approach with a skin incision of 8-10 cm, the conjoint tendon and the pectoralis major are retracted medially, while the deltoid muscle laterally. The long head of the biceps (LHBs) and the fracture are identified. Fracture hematoma as well as any subdeltoideus scar tissue is removed. The fracture is reduced using the LHB as an anatomical landmark to restore the correct rotation and length of the bone (Fig. 2) (Video 1).

**Figure 3:**
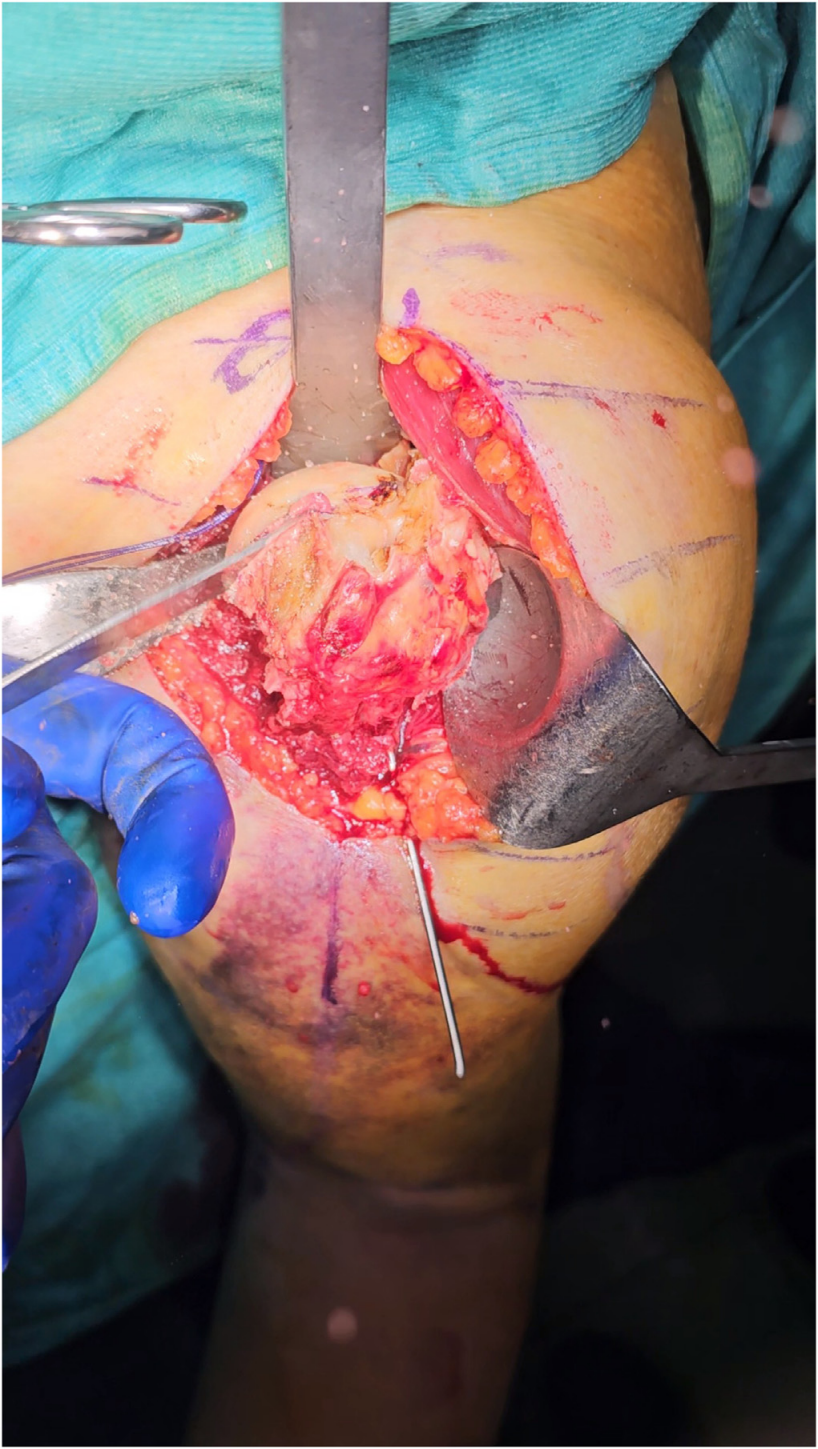
A small resection of the HH is performed with the oscillating saw, preferably in a 0° rotation of the arm. *HH*, humeral head.

**Figure 4:**
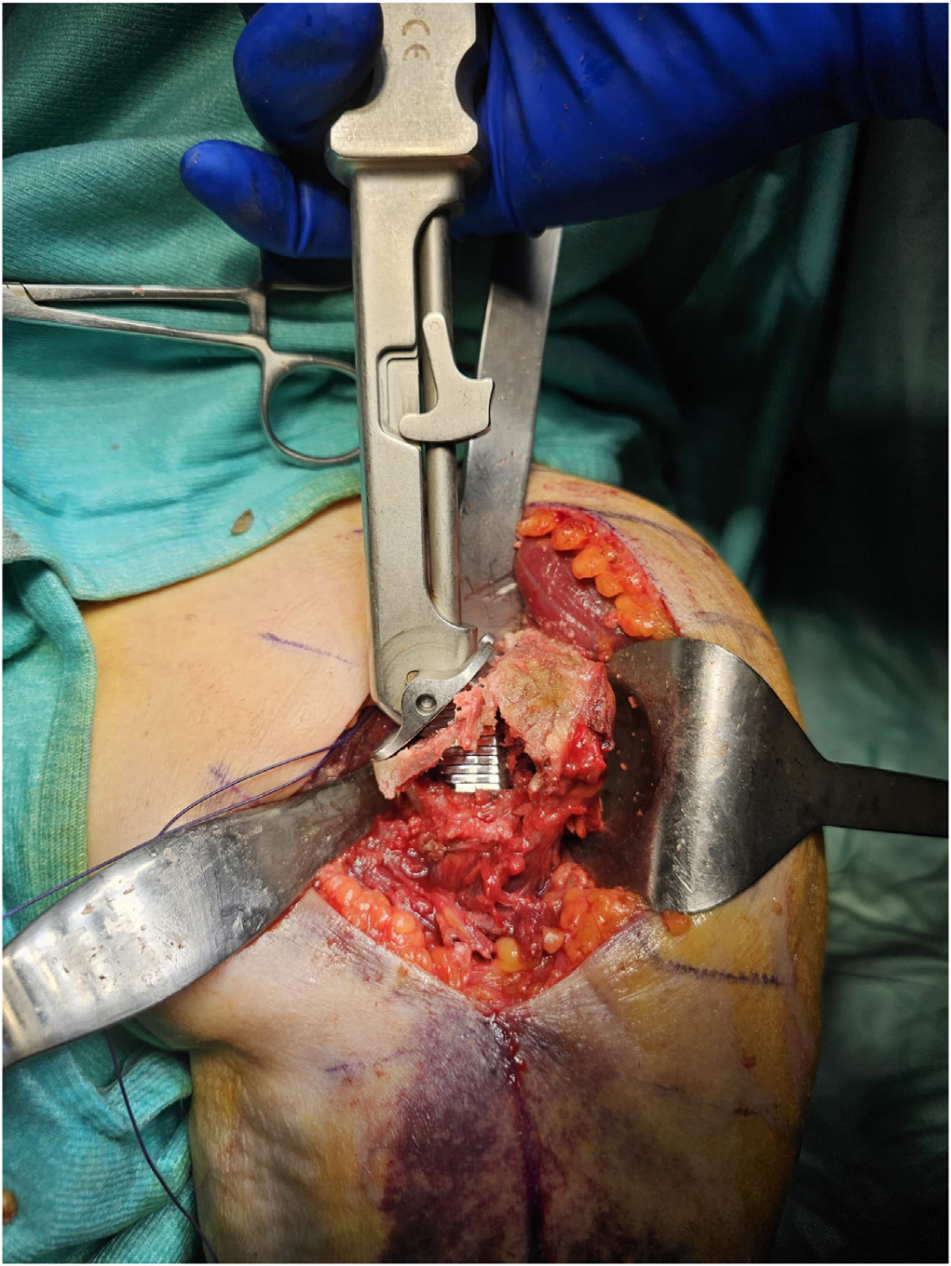
The implant’s broaches serve to prepare the canal and give axial compression to the fragments that will occupy the metaphyseal portion.

The subscapularis’ inferior vessels should be cauterized and a LHB tenotomy/tenodesis could be performed. The supraspinatus tendon, if intact, is removed, to reduce the forces cranially dislocating the proximal humerus. The insertion of the subscapularis is then identified, medially to the LHB, and the tendon is loaded with nonabsorbable sutures. It is important to underline that, since the rotation of the arm does not move the proximal humerus because of the fracture, the subscapularis sutures could be pulled to externally rotate the HH. Subscapularis peeling with complete release of the tendon from the small tuberosity allows exposure of the joint. Inferior capsule is detached from the humeral calcar and the HH is dislocated together with the diaphysis. One or Two Kirschner-wires could be used to temporary stabilize the SN fracture during HH exposure and dislocation to avoid any significative displacement of the fragments. In any case, we prefer to keep the arm in 0° of rotation during the shaft preparation to reduce any forces displacing the fracture. With an oscillating saw, a thin HH resection is performed, either using an extramedullary cutting guide or free hand (Fig. 3). The diaphyseal canal is then identified and progressively broached using the reamer as an endomedullary nail with the desired angle of retroversion (Fig. 4). Before broaching, the sutures passed into the posterior rotator cuff could be used to pull the metaphyseal fragments on the diaphysis, while the arm could be angulated or rotated to reduce the shaft under the proximal part of the humerus. Care should be taken to avoid bone separation between calcar fragment and the GT especially in case of a high fracture line (as showed in the surgical technique video), or in case of severe osteopenic bone. However in case of metaphyseal intraoperative fracture, bone sutures and autograft from the resected HH could be used to keep the two fragments united. In this scenario, the absence of the subscapularis force will reduce the tendency of the calcar fragment to dislocate medially, preventing displacement in confront to the GT.

To avoid excessive cancellous bone removal from the metaphysis and to use the humeral tray to compress the metaphysis on the shaft, we prefer to use an onlay stem design. For the same reason, we prefer to undersize the humeral stem, using the smallest size that avoids rotation of the humeral component as definitive size, and eventually adding autologous cancellous bone graft to improve stem stability. A trial component is positioned to protect the humerus and the glenoid is then exposed. The glenoid component is implanted in standard fashion.

**Figure 5:**
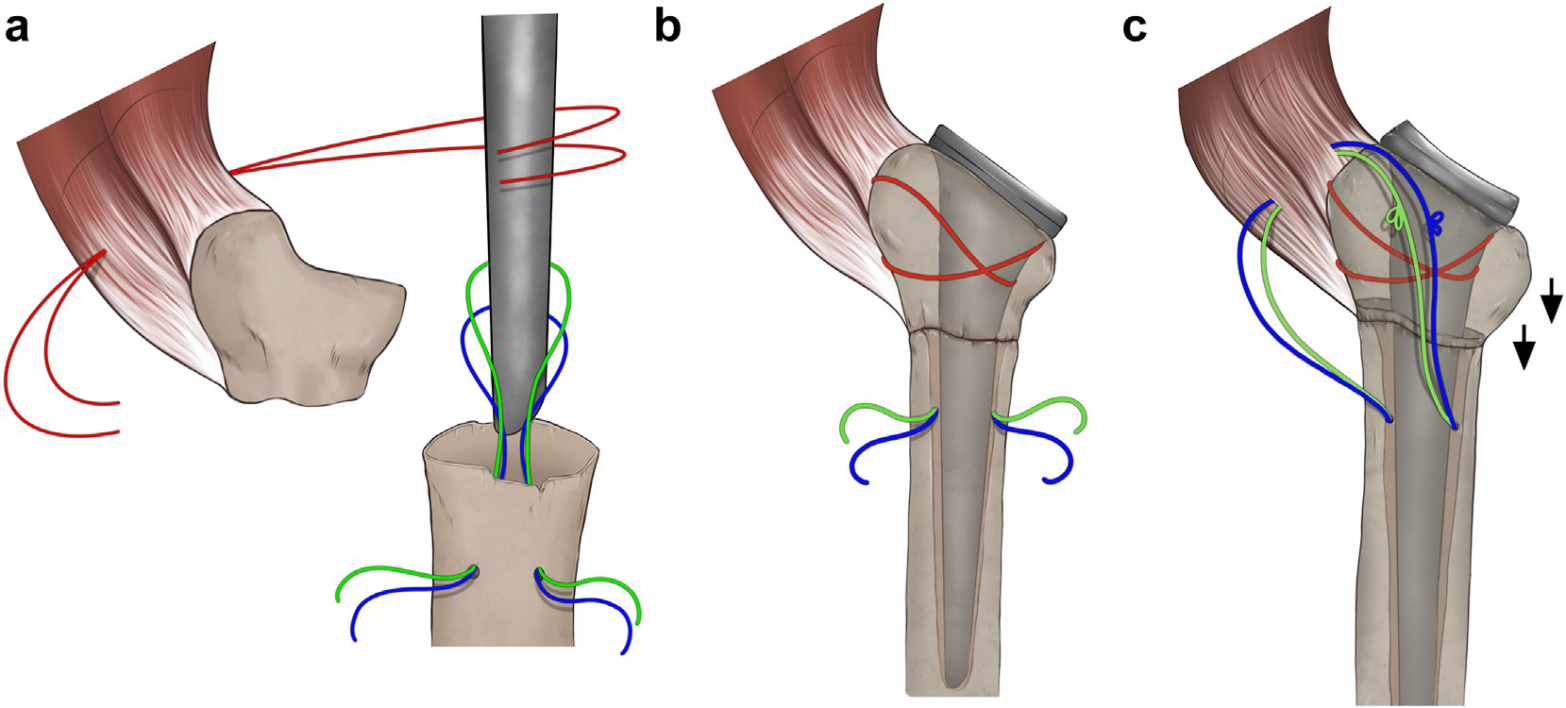
A schematic representation of the suture configuration during stem placement (**a**), epiphysis reduction aided by tuberosity suture cerclage (**b**) and metaphysis suture tying with vector of reduction and fixation (**c**). The arrows represent, intuitively, the vector of reduction and compression of the proximal fragment given by the suture cerclage.

**Figure 6:**
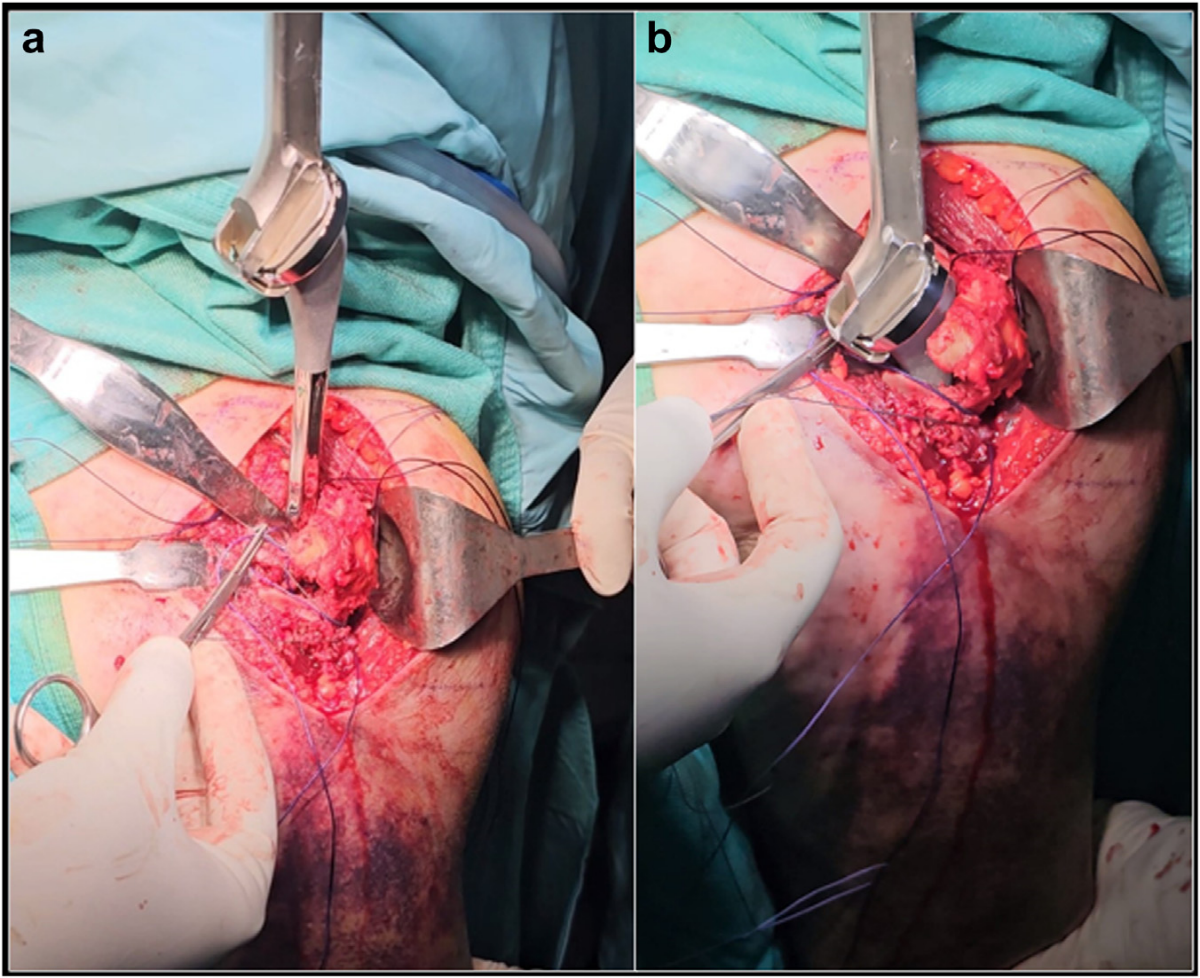
The stem is inserted inside the previously prepared loop of diaphyseal sutures (**a**), and two additional nonresorbable sutures are passed alongside the implant’s neck (**b**).

The trial humeral metaphysis and liner are inserted, and prosthetic reduction is obtained. Once the stability maneuvers are satisfactory, the prosthesis is dislocated again and humeral trial stem is removed. Multiple transosseous nonabsorbable sutures are passed through the lateral cortex of the diaphysis and in the proximal metaphyseal fragment at the level of the infraspinatus tendon insertion (Fig. 5). Cancellous bone from the HH could be used both in the endomedullary canal and in the fracture to enhance the stem osteointegration and fracture healing. The definitive uncemented stem is implanted, taking care to introduce it inside a loop of the diaphysis sutures, and to associate two nonresorbable sutures along the prosthetic neck (Fig. 6).

**Figure 7:**
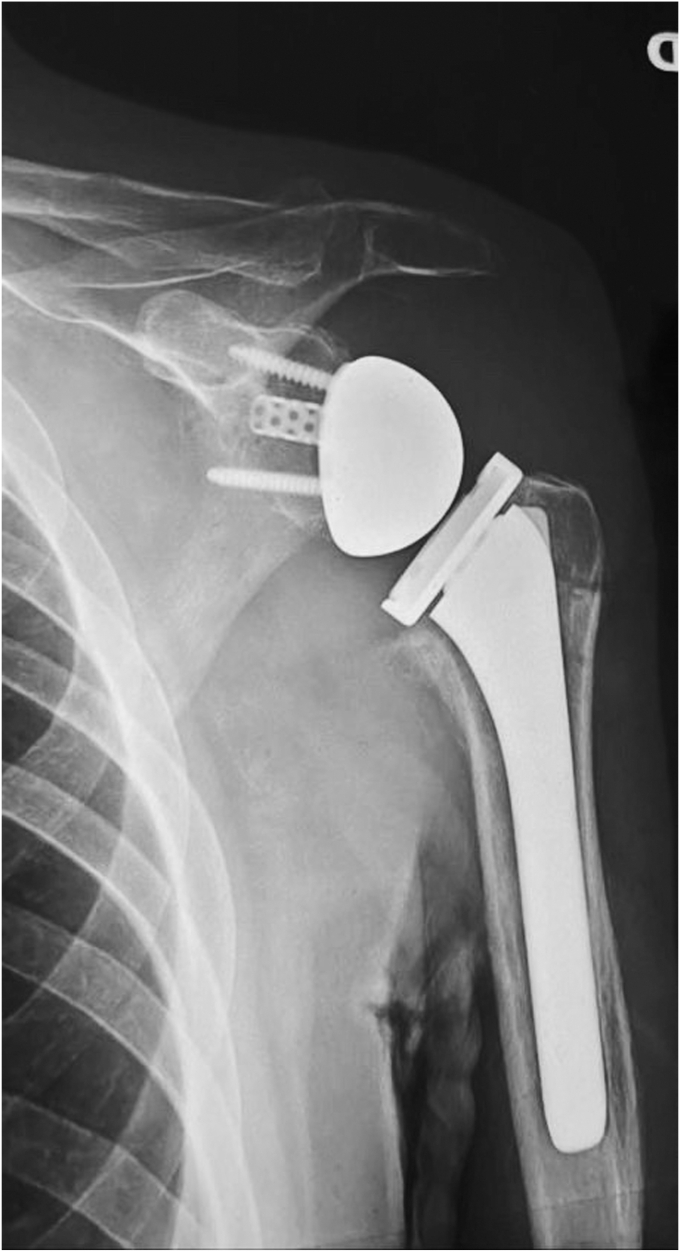
The correspondent postoperative X-Ray showing reduction of fracture site and alignment of the prosthesis with an uncemented stem.

The prosthesis is reduced. The humeral stem works like an endomedullary nail, stabilizing the fracture in the coronal and sagittal plain (Fig. 7). The diaphyseal, transtendon and circular periprosthetic sutures are used to ensure the rotational stability of the humeral metaphysis to the diaphysis and the prosthesis. We prefer not to reattach the subscapularis tendon to reduce any force which could displace the SN fracture and the risk of stiffness.

Finally, the deltopectoral approach is sutured after multiple lavages, hemostasis checks, and redon placement.

## Rehabilitation protocol

The operated arm is positioned in a neutral rotation brace for three weeks. Passive and active elbow and hand mobilization should be encouraged from the first postoperative day, but passive shoulder mobilization is allowed after twenty days to avoid the development of pain and to protect fracture healing. We suggest starting the restoration of passive elevation in warm water for at least one month, three times per week in association with daily autonomous exercises. Thereafter, the water exercises should be alternated with passive mobilization facilitated by a therapist and eccentric exercises for elevation should be initiated. Active muscular strengthening against resistance usually starts at three months when the passive range of motion (ROM) is restored.

## Expected outcomes

There is a paucity of studies about two parts PHF. Launonen et al^18^ conducted a multicentric semiblinded randomized controlled clinical trial to compare the clinical outcome of surgical treatment, with plate and screw fixation, and conservative treatment in displaced two parts fractures in the elderly (>60 years old). The authors observed no significant differences in clinical outcomes at two years between surgery and nonoperative treatment. A recent randomized controlled clinical trial,^10^ comparing nail versus plate fixation in two-parts PHFs, did not find statistically significant differences between the two types of treatment.

The “shish-kebab” technique has already been described to treat type 3 PHF sequelae, with reported favorable outcome (better active forward elevation and SSV) due to GT preservation.^2^^,^^3^

In our opinion, the “shish-kebab” technique is applicable to PHFs as well and, in comparison with the standard technique, has a series of important advantages. First, humeral stem implantation does not require a tuberosity osteotomy. Indeed, in the standard technique the surgeon turns a two-parts fracture into a four-parts one, to remove the HH and to insert the humeral stem. After the implantation, cortical pieces of the tuberosities are then synthetized with nonabsorbable sutures. The importance of tuberosities healing has been demonstrated in different papers which confirmed that tuberosities resorption and migration are associated with poorer clinical outcomes and subjective results.^1^^,^^13^ Several techniques have been proposed to improve tuberosities healing,^4^^,^^8^^,^^25^ but a gold standard is still far to be found, and tuberosity healing rate reported in the literature ranges from 40% up to 84%.^1^ Our thesis is that the preservation of a single metaphyseal bone fragment could improve tuberosities healing and reduce the risk of tuberosity migration. Indeed, with the “shish-kebab” technique, the dislocating forces of the posterior rotator cuff on the GT are counterbalanced not only by the suture fixation, but also by the humeral stem and by the integrity of the anterior and posterior humeral cortex. Moreover, the absence of the subscapularis could reduce the forces dislocating the LT and improve shoulder ROM.

A second advantage of this technique is an easier intraoperative evaluation of stem height with a standardized bone marker (GT). Previous studies reported that humeral lengthening is correlated to improved active elevation, while humeral shortening is a risk factor for instability.^16^ However, Henninger et al^11^ showed that, for each 3 mm increase in implant thickness, there is a corresponding increase in deltoid tension and in loss of adduction. This balance between stability and ROM is the key for a successful RSA and, with our technique without tuberosity osteotomy, after fracture reduction the surgeon has valuable anatomical landmarks to adjust humeral stem height to best fit the anatomy of the patient.^23^

## Complications

A major potential complication of this technique could be the bone resorption of the proximal humerus. In previous studies on RSA for three and four-parts fracture, GT resorption was observed between 10% and 20% of the cases.^6^^,^^26^ However, we believe that the retaining of a large metaphyseal bone fragment will grant greater healing potential in comparison with isolated cortical bone fragments resulting from the osteotomy of the tuberosities.

Instability is another possible complication, related to the stem height and muscle function. It is reported in 1-5% of the cases.^23^ Theoretically, the subscapularis’ peeling could increase the risk of prosthesis anterior dislocation, but we believe that the correct deltoid tensioning, the preserved height GT on the lateral cortex, the use of an onlay stem with an increased deltoid wrapping, could stabilize the joint even without the integrity of the subscapularis.

Aseptic humeral stem loosening is an uncommon complication following RSA. In 2011, a systematic review on RSA complications, with almost 10 years of follow-up, showed a rate of humeral subsidence of 1.3%.^31^ In the same year, a multicenter study reported that the rate of stem loosening was almost double for cemented stems (11.8%) in confront with uncemented stems (5.9%).^19^ In 2022, Rossi et al found no significative difference, at medium term follow-up (41 months), for humeral stem loosening in the subgroup of PHFs treated with cemented or uncemented stem.^24^ On the contrary, humeral stress shielding is more frequently reported, especially in uncemented humeral stem, probably because of the stem oversize used to stabilize the humeral component in case of metaphyseal bone loss.^15^ However, it has been reported that the use of undersized stem with cancellous bone autograft could reduce the incidence of stress shielding.^14^ For this reason, in case of stem rotational instability, we suggest to eventually cement the tip of the prosthesis in the endomedullary canal, rather than increase the stem size.

Other complications as infection, intraoperative fractures, glenoid loosening, acromion and scapular spine fracture are not influenced by the specific technique described in the paper and are associated with the use of RSA in PHF independently from the surgical approach.

## Conclusion

The “shish-kebab” technique can be a valuable tool for the orthopedic surgeon who has to deal with a two-part PHF through reverse shoulder prosthesis. Its application, following the correct step-to-step procedure, is fast and effective and leads to reliable reduction of the fragments and fixation of the fracture aided by sutures and an endomedullary nail-like effect of the stem. The technique must be applied with a thorough understanding of the fracture’s morphology and personality to be treated, and knowledge of the key points of performing a RSA over a fracture to avoid complications.

## Disclaimers:

Funding: No funding was disclosed by the authors.

Conflicts of interest: The authors, their immediate families, and any research foundation with which they are affiliated have not received any financial payments or other benefits from any commercial entity related to the subject of this article.
